# Reduced ER-mitochondrial contact sites and mitochondrial Ca^2+^ flux in *PRKN*-mutant patient tyrosine hydroxylase reporter iPSC lines

**DOI:** 10.3389/fcell.2023.1171440

**Published:** 2023-09-08

**Authors:** Mutsumi Yokota, Yutaro Yoshino, Mitsuko Hosoi, Ryota Hashimoto, Soichiro Kakuta, Takahiro Shiga, Kei-Ichi Ishikawa, Hideyuki Okano, Nobutaka Hattori, Wado Akamatsu, Masato Koike

**Affiliations:** ^1^ Department of Cell Biology and Neuroscience, Juntendo University Graduate School of Medicine, Tokyo, Japan; ^2^ Laboratory of Cell Biology, Biomedical Research Core Facilities, Juntendo University Graduate School of Medicine, Tokyo, Japan; ^3^ Laboratory of Morphology and Image Analysis, Biomedical Research Core Facilities, Juntendo University Graduate School of Medicine, Tokyo, Japan; ^4^ Center for Genomic and Regenerative Medicine, Juntendo University Graduate School of Medicine, Tokyo, Japan; ^5^ Department of Neurology, Juntendo University School of Medicine, Tokyo, Japan; ^6^ Department of Physiology, Keio University School of Medicine, Tokyo, Japan; ^7^ Neurodegenerative Disorders Collaborative Laboratory, RIKEN Center for Brain Science, Saitama, Japan

**Keywords:** *PRKN*, iPSC, tyrosine hydroxylase reporter, dopaminergic neurons, ER-mitochondrial contact sites

## Abstract

Endoplasmic reticulum-mitochondrial contact sites (ERMCS) play an important role in mitochondrial dynamics, calcium signaling, and autophagy. Disruption of the ERMCS has been linked to several neurodegenerative diseases, including Alzheimer’s disease (AD), Parkinson’s disease (PD), and amyotrophic lateral sclerosis (ALS). However, the etiological role of ERMCS in these diseases remains unclear. We previously established tyrosine hydroxylase reporter (*TH-*GFP) iPSC lines from a PD patient with a *PRKN* mutation to perform correlative light-electron microscopy (CLEM) analysis and live cell imaging in GFP-expressing dopaminergic neurons. Here, we analyzed ERMCS in GFP-expressing *PRKN-*mutant dopaminergic neurons from patients using CLEM and a proximity ligation assay (PLA). The PLA showed that the ERMCS were significantly reduced in *PRKN-*mutant patient dopaminergic neurons compared to the control under normal conditions. The reduction of the ERMCS in *PRKN*-mutant patient dopaminergic neurons was further enhanced by treatment with a mitochondrial uncoupler. In addition, mitochondrial calcium imaging showed that mitochondrial Ca^2+^ flux was significantly reduced in *PRKN*-mutant patient dopaminergic neurons compared to the control. These results suggest a defect in calcium flux from ER to mitochondria is due to the decreased ERMCS in *PRKN*-mutant patient dopaminergic neurons. Our study of ERMCS using *TH-*GFP iPSC lines would contribute to further understanding of the mechanisms of dopaminergic neuron degeneration in patients with *PRKN* mutations.

## Introduction

Mitochondria play an essential role in the regulation of cellular energy metabolism, redox signaling, and apoptosis. The endoplasmic reticulum-mitochondrial contact sites (ERMCS) have been shown to be associated with a variety of cellular processes, such as lipid biosynthesis, mitochondrial dynamics, calcium signaling, and autophagy (Rowland and Voeltz, 2012; Paillusson et al., 2016; Csordás et al., 2018; Gómez-Suaga et al., 2018). Mitochondrial abnormalities, including disruption of ERMCS, have been suggested to be involved in the pathogenesis of neurodegenerative diseases, such as Alzheimer’s disease (AD), Parkinson’s disease (PD), and amyotrophic lateral sclerosis (ALS) (Paillusson et al., 2016; Okano and Morimoto, 2022). For example, a reduction in ER-mitochondria associations in PD patients (Paillusson et al., 2017; Liu et al., 2019) and the occasional localization of PD-causing proteins at ERMCS (Ottolini et al., 2013; Guardia-Laguarta et al., 2014; Gelmetti et al., 2017) have been reported. The expression of ERMCS-associated proteins has been shown to be altered in AD brain and AD mouse models (Hedskog et al., 2013). ALS model mice have exhibited impaired ultrastructure of the ERMCS in motor neurons (Watanabe et al., 2016). However, the physiological significance of ERMCS in these diseases has not been elucidated in detail.

The Parkin RBR E3 ubiquitin protein ligase is encoded by the *PRKN/PARK2* gene, which is the most common causative gene for the autosomal recessive form of PD. Parkin is a key component of mitochondrial quality control mechanisms (Abou-Sleiman et al., 2006; Narendra et al., 2008; Scarffe et al., 2014). PD patients with *PRKN* mutations show preferential degeneration of dopaminergic neurons in the substantia nigra pars compacta. Several studies using patient fibroblasts from patients with *PRKN* mutations have reported ERMCS alteration (Gautier et al., 2016; Basso et al., 2018), but just one study (McLelland et al., 2018) has reported ERMCS abnormalities using dopaminergic neurons derived from *PRKN*-mutant patient iPSCs. It is still unclear how ERMCS are involved in the cell death of dopaminergic neurons in *PRKN*-related PD.

We have previously established tyrosine hydroxylase reporter (*TH-*GFP) iPSC lines from two control subjects and one PD patient with a *PRKN* mutation (Yokota et al., 2021). The *TH*-GFP iPSC lines express GFP specifically in dopaminergic neurons, making them suitable for correlative light-electron microscopy (CLEM) analysis and live cell imaging of GFP-expressing dopaminergic neurons (Yokota et al., 2021). We have shown that small and low-functional mitochondria unique to dopaminergic neurons and the preferential death of *PRKN*-mutant patient dopaminergic neurons under 30 μM a mitochondrial uncoupler, carbonyl cyanide m-chlorophenyl hydrazine (CCCP) for 24 h (Yokota et al., 2021). Meanwhile, we have not yet analyzed ERMCS in *PRKN*-mutant patient dopaminergic neurons derived from *TH*-GFP iPSC lines.

In this study, ERMCS were observed in GFP-positive dopaminergic neurons derived from *TH-*GFP iPSC lines using CLEM and proximity ligation assay (PLA) to clarify the alterations of ERMCS in *PRKN*-mutant dopaminergic neurons. The PLA suggest that ERMCS were significantly reduced in *PRKN*-mutant patient lines compared to that in the control lines. In addition, mitochondrial calcium imaging provided evidence that mitochondrial Ca^2+^ flux was decreased in the *PRKN*-mutant patient lines. These results suggest that dopaminergic neurons from *PRKN*-mutant patients have impaired calcium flux from the ER to the mitochondria due to reduced ERMCS. Our findings would partially help us to understand the mechanisms of dopaminergic neuron degeneration in patients with *PRKN* mutations.

## Methods

### Human iPSCs

The four *TH*-GFP iPSC lines used for CLEM, PLA, and Ca^2+^ imaging were previously established from the following original iPSC lines (Yokota et al., 2021). 201B7, as a control line, was kindly provided by Dr. Shinya Yamanaka at Kyoto University (Takahashi et al., 2007). The other two control lines (WD39 and eKA4), and two PD patient lines with a *PRKN* mutation (PA9, exon 2-4 homozygous deletions and PB2, exon 6, 7 homozygous deletions) were established by H.O. (Imaizumi et al., 2012; Matsumoto et al., 2016). The *PRKN* knock-in/knock-out iPSC line (B7PA21) was established by Dr. Minoru Narita at Hoshi University School of Pharmacy and Pharmaceutical Science in a previous report (Kuzumaki et al., 2019). One control iPSC (JB6) and one PD patient iPSC with *PRKN* mutation (PH13, exon 3 homozygous deletion) were generated from peripheral blood mononuclear cells with CytoTune™-iPS 2.0 Sendai Reprogramming Kit (Nacalai) encoding the four factors (Oct3/4, Sox2, Klf4, and c-Myc) using the same method as in previous report (Ishikawa et al., 2022). The use of human iPSCs was approved by the Ethical Committees of Juntendo University School of Medicine (Approval Number 2017032).

### Differentiation of the iPSC lines into dopaminergic neurons

The iPSC lines were cultured on plates coated with iMatrix-511 (Nippi) and expanded in StemFit AK02N medium (Ajinomoto). The iPSC lines were differentiated into dopaminergic neurons using a previously established direct neurosphere converting method (Imaizumi et al., 2015; Matsumoto et al., 2016; Fujimori et al., 2017; Yamaguchi et al., 2020). For mitochondrial stress, differentiated cells were treated with 30 μM CCCP (Sigma-Aldrich) for 24 h before fixation.

### Correlative light-electron microscopy

For CLEM analysis, dissociated neurospheres were reseeded on gridded coverslips (Matsunami) after coating with poly-L-ornithine and fibronectin. On day 7–10 of culture, cells were fixed in 2% paraformaldehyde, 0.5% glutaraldehyde, and 50 mM sucrose in 0.1 M phosphate buffer. Brightfield and fluorescence images were captured using a BZ-X710 fluorescence microscope (Keyence). Next, cells were fixed in 2% glutaraldehyde and 50 mM sucrose in 0.1 M phosphate buffer, followed by post-fixation with 1% osmium tetroxide. Fixed cells were dehydrated and embedded in Epon812 (Oken Shoji). Ultrathin sections were cut using an ultramicrotome UC6 (Leica), stained with uranyl acetate and lead citrate, and examined using a Hitachi HT7700 electron microscope (Hitachi). The electron microscope images were analyzed using ImageJ software (https://imagej.nih.gov/ij/).

### Proximity ligation assay

PLA was performed using Duolink PLA (Sigma-Aldrich) to quantify ER-mitochondria interactions. On day 7–10 of culture, after reseeding dissociated neurospheres, differentiated cells were fixed with 4% paraformaldehyde in PBS for 10 min and permeabilized with 0.1% Triton X-100 in PBS for 15 min at room temperature. Cells were then blocked with Duolink Blocking Solution for 1 h at 37°C and stained with primary antibodies for 1 h at 37°C. The following antibodies were used: rat anti-GFP antibody (MBL; 1:500) for *TH*-GFP iPSC lines or sheep anti-TH antibody (PelFreez; 1:400) for other iPSC lines, rabbit anti-VDAC antibody (Proteintech; 1:500), and mouse anti-IP3R3 antibody (Santa Cruz Biotechnology; 1:100). Cells were then washed and stained with Alexa Fluor 488-conjugated donkey anti-rat secondary antibody (Jackson ImmunoResearch; 1:400) for *TH*-GFP iPSC lines or donkey anti-sheep secondary antibody (Thermo Fisher Scientific; 1:800) for other iPSC lines, DAPI (Thermo Fisher Scientific; 1:10,000), and Duolink PLA Probe anti-mouse PLUS and anti-rabbit MINUS for 1 h at 37 °C. PLA signals were detected using the Duolink Detection Reagents FarRed. PLA fluorescence images were captured using a confocal microscope (Zeiss LSM880 with Airyscan). Airyscan z-stack images were processed using a maximum-intensity-projection.

### Immunofluorescence staining

On day 7–10 of culture, after reseeding dissociated neurospheres, differentiated cells were fixed with 4% paraformaldehyde in PBS for 10 min and permeabilized with 0.1% Triton X-100 in PBS for 5 min at room temperature. Cells were then blocked with 2% BSA in PBS for 30 min and stained with primary antibodies for 2 h at room temperature. The following antibodies were used: rat anti-GFP antibody (MBL; 1:500), mouse anti-Tom20 antibody (Santa Cruz Biotechnology; 1:100), and rabbit anti-Calnexin antibody (Abcam; 1:1,000). Cells were then washed and stained with Cy3-, Alexa Fluor 488-, or 647-conjugated donkey secondary antibodies (Jackson ImmunoResearch; 1:400 or Thermo Fisher Scientific; 1:800) and DAPI (Thermo Fisher Scientific; 1:10,000) for 1 h at room temperature. Immunostaining images were taken using a confocal microscope (Zeiss LSM880). Airyscan images were processed using an Airyscan-processing. The fluorescence images were analyzed using a Threshold tool in ImageJ software (https://imagej.nih.gov/ij/) for selection of the staining positive area.

### Mitochondrial and cytosolic Ca^2+^ imaging

The pLV-EF1α-R-CEPIA3*mt* containing the R-CEPIA3*mt* ORF (Addgene plasmid #140464) (Kanemal et al., 2020) was synthesized using Vector Builder. The pLV-EF1α-R-CEPIA3*mt* and the packaging plasmids were co-transfected into 293T cells using TransIT-293 Reagent (Mirus Bio), and then the supernatants were concentrated using a Lenti-X Concentrator (Clontech) after 4 days of transfection. On day 1 of culture after reseeding dissociated neurospheres derived from the *TH-*GFP iPSCs, the cells were infected with the concentrated lentivirus at a rate of 1 in 250. On day 8 of culture, the cells were perfused with HBSS and the fluorescence intensity of R-CEPIA3*mt* was measured via an objective lens (PlanApo ×60, N.A. 1.20; Olympus) and a cooled CCD camera (ORCA-ER; Hamamatsu Photonics) every 1 s for 80 s using Aquacosmos 2.0 (336 × 256 resolution) (Hamamatsu Photonics) for mitochondrial Ca^2+^ imaging. For cytosolic Ca^2+^ imaging, the cells were incubated with 5 μM Fura 2-acetoxymethyl ester (AM) (Dojindo) and 0.01% Pluronic F-127 (Biotium) in HBSS for 30 min at room temperature and perfused with HBSS, and the Fura 2-AM fluorescence ratio (F_340_/F_380_) was measured via an objective lens (S Fluor ×40, N.A. 0.90; Nikon) and a cooled CCD camera (ORCA-ER) every 1 s for 80 s using Aquacosmos 2.0 (336 × 256 resolution) (Hamamatsu Photonics). Next, the cells were stimulated by perfusion with 10 μM histamine (Wako), the inositol triphosphate-generating agonist, and the fluorescence intensity of R-CEPIA3*mt* or the Fura 2-AM fluorescence ratio was measured every 1s for 300 s. The ratio of the change in fluorescence intensity was determined using the following equation. ΔF/F_0_ = (fluorescence intensity—minimum fluorescence intensity before stimulation)/minimum fluorescence intensity before stimulation. The Fura 2-AM fluorescence was expressed as ratiometric value (R: F_340_/F_380_). The localization of R-CEPIA3*mt* to mitochondria in the differentiated cells was confirmed by staining for 30 min with 50 nM Mitotracker Deep Red (Thermo Fisher Scientific). Live cell images were taken using a confocal microscope (Zeiss LSM880).

### Statistical analysis

GraphPad Prism 8 software was used for statistical analyses. Differences between groups were evaluated using the unpaired *t*-test or two-way ANOVA with Bonferroni’s multiple comparison test, as appropriate. Statistical significance was set at *p* < 0.05.

## Results

### CLEM analysis for the ERMCS in GFP-positive dopaminergic neurons derived from *TH*-GFP iPSC lines

**FIGURE 1:**
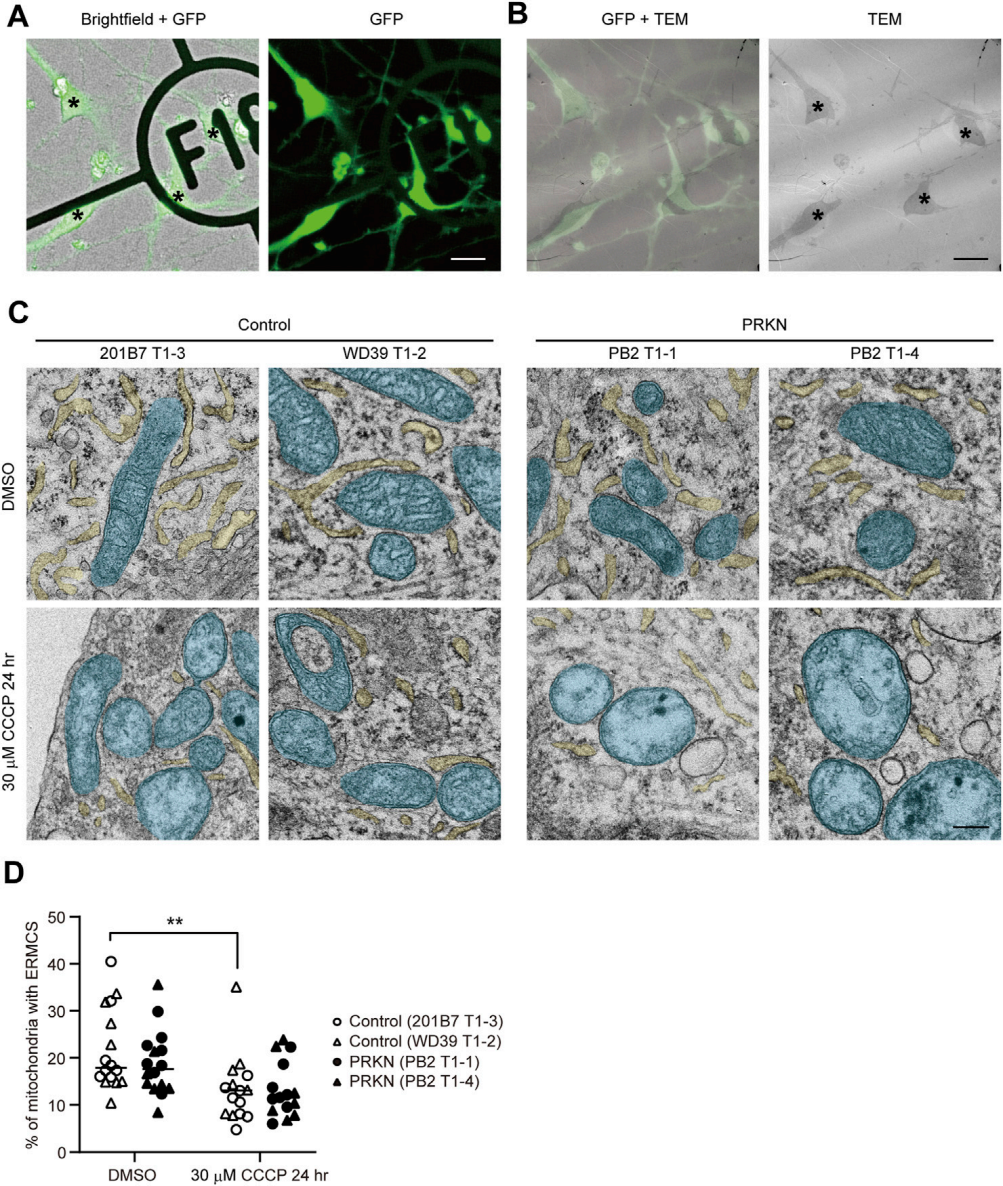
CLEM analysis of GFP-positive dopaminergic neurons derived from *TH-*GFP iPSCs. **(A)** The merged brightfield and GFP (left) and GFP (right) images of *TH-*GFP iPSC-derived cells on the gridded coverslips. Asterisks indicate GFP-positive dopaminergic neurons. Scale bar, 20 µm. **(B)** The merged GFP and TEM (left) and TEM (right) images of *TH*-GFP iPSC-derived cells in the same location as **(A)** in the ultrathin sections. Asterisks indicate GFP-positive dopaminergic neurons. Scale bar, 20 µm. **(C)** Representative TEM images of GFP-positive dopaminergic neurons under normal conditions (DMSO treatment, top) and 30 μM CCCP treatment for 24 h (bottom) derived control and *PRKN*-mutant patient *TH*-GFP iPSCs. ER and mitochondria are painted light yellow and cyan, respectively. “PRKN” represents *PRKN*-mutant patient. Scale bar, 200 nm. **(D)** Quantitative analysis of ERMCS in GFP-positive dopaminergic neurons from EM images. The graph represents the ratio of mitochondrial length in contact with ER (<30 nm) to the total mitochondrial circumference in dopaminergic neurons (DMSO control; n = 16, DMSO PRKN; n = 16, CCCP control; n = 15, CCCP PRKN; n = 15). “PRKN” represents *PRKN*-mutant patient. Horizontal lines indicate median values. Statistical significance was evaluated using the two-way ANOVA with Bonferroni’s multiple comparison test. **p < 0.01. There was no significant difference in ERMCS between DMSO and CCCP in *PRKN*-mutant dopaminergic neurons (*p* = 0.0644). There was no significant difference in ERMCS between control and *PRKN*-mutant patient lines under normal conditions (*p* = 0.5508) and CCCP treatment (*p* > 0.9999).

To examine the ER-mitochondria interface in control and *PRKN*-mutant patient dopaminergic neurons, we differentiated into dopaminergic neurons derived from two control *TH*-GFP iPSC lines (201B7 T1-3 and WD39 T1-2) and two *PRKN*-mutant patient *TH*-GFP iPSC lines (PB2 T1-1 and PB2 T1-4) (Yokota et al., 2021) using the direct neurosphere conversion method (Imaizumi et al., 2015; Matsumoto et al., 2016; Fujimori et al., 2017; Yamaguchi et al., 2020). We treated the differentiated cells with 30 μM CCCP for 24 h to investigate the change in the ER-mitochondria interface in dopaminergic neurons under pathological conditions, because several studies have reported that 30 μM CCCP treatment for 24–48 h exhibited pathogenicity specific to patient dopaminergic neurons (Imaizumi et al., 2012; Suzuki et al., 2017; Yamaguchi et al., 2020; Yokota et al., 2021). We acquired brightfield and fluorescence images of the differentiated cells on gridded coverslips to determine the location of GFP-positive dopaminergic neurons, and then fixed them strongly for electron microscopy using the CLEM method described previously (Yokota et al., 2021) (Figure 1A). We searched for GFP-positive cells on ultrathin sections using cell shapes and positions in the brightfield and fluorescence images and observed the ER tubules around mitochondria in GFP-positive cells under a transmission electron microscope (Figure 1B).

CLEM analysis of GFP-positive dopaminergic neurons revealed that the CCCP treatment significantly reduced the ER around mitochondria under CCCP treatment in control lines (Figures 1C, D; *p* = 0.0042). Comparing control and *PRKN*-mutant patient lines, *PRKN*-mutant patient dopaminergic neurons tended to have less ERMCS under normal conditions, but this difference was not statistically significant (Figures 1C, D; *p* = 0.5508). CLEM analysis using *TH*-GFP iPSCs indicate that dopaminergic neurons under CCCP treatment tend to have less ERMCS than dopaminergic neurons under normal conditions.

### PLA for quantification of the ERMCS in dopaminergic neurons derived from *TH*-GFP iPSC lines

**FIGURE 2:**
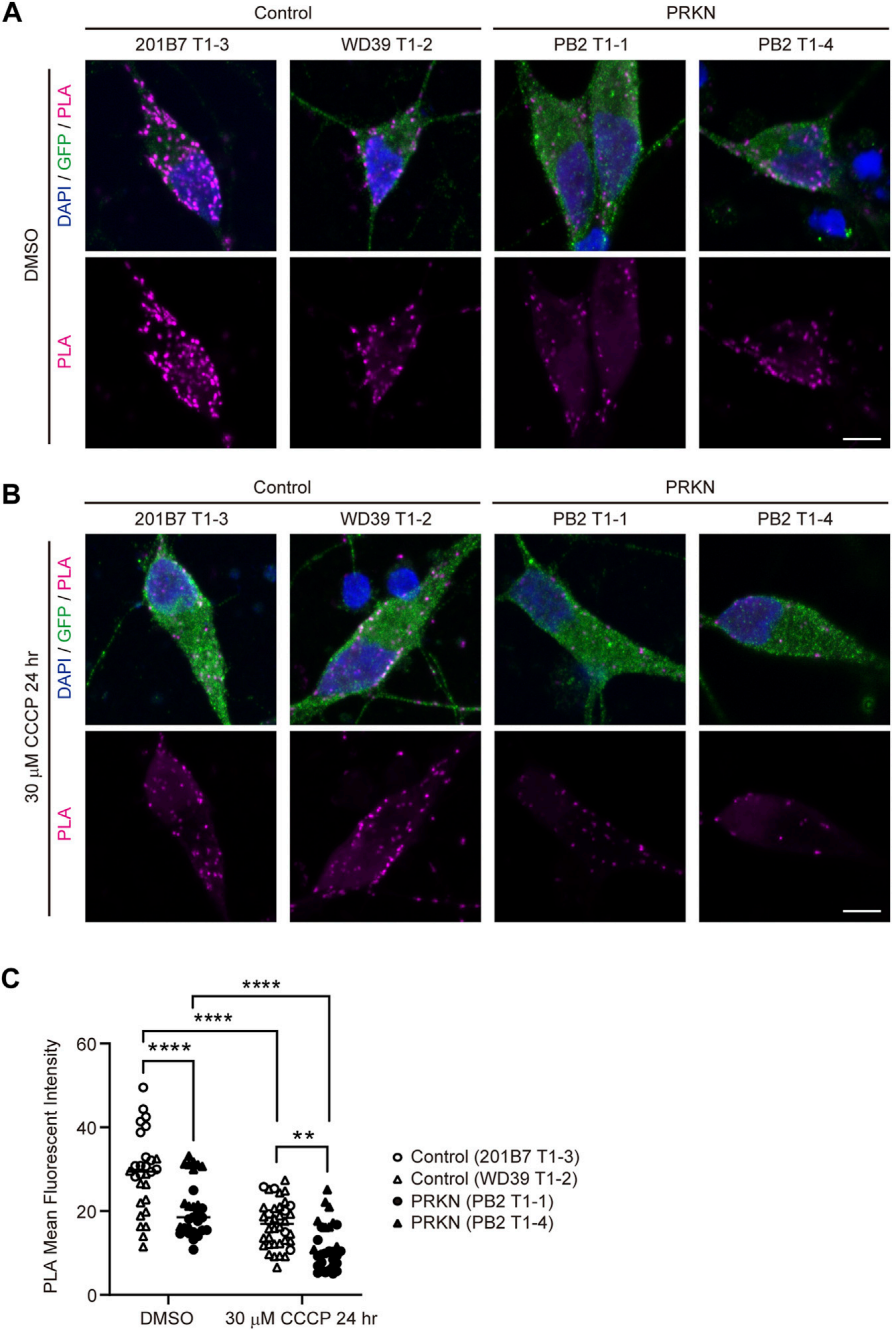
PLA in dopaminergic neurons derived from *TH-*GFP iPSCs. **(A)** Representative PLA images of GFP-positive dopaminergic neurons under normal conditions (DMSO treatment) derived from control and *PRKN*-mutant patient *TH*-GFP iPSCs. PLA signals represent the interactions between VDAC and IP3R3. “PRKN” represents *PRKN*-mutant patient. Scale bar, 5 µm. **(B)** Representative PLA images of GFP-positive dopaminergic neurons under 30 μM CCCP treatment for 24 h derived from control and *PRKN*-mutant patient *TH-*GFP iPSCs. PLA signals represent the interactions between VDAC and IP3R3. “PRKN” represents *PRKN*-mutant patient. Scale bar, 5 µm. **(C)** Quantitative analysis of the MFI of PLA signals in GFP-positive dopaminergic neurons in PLA images. The MFI was measured by surrounding the cell body region of GFP-positive cells (DMSO control; n = 28, DMSO PRKN; n = 29, CCCP control; n = 38, CCCP PRKN; n = 30) in ZEN software. “PRKN” represents *PRKN*-mutant patient. Horizontal lines indicate median values. Statistical significance was evaluated using the two-way ANOVA with Bonferroni’s multiple comparisons test. **p < 0.01, ****p < 0.0001.

We thought that the EM analysis in ultrathin sections was not sufficient to compare ERMCS between control and patient lines, and we therefore used the *in situ* proximity ligation assay (PLA) and captured PLA fluorescence z-stack images using a confocal microscope to quantify ERMCS in whole dopaminergic neuronal soma. In PLA, fluorescence signals are only emitted when two target proteins are in close proximity within 40 nm of each other (Söderberg et al., 2006). In our study, PLA was performed using antibodies against VDAC and IP3R3, which are markers for the mitochondrial outer membrane and ER, respectively. PLA in dopaminergic neurons revealed that 30 μM CCCP treatment for 24 h remarkably reduced the VDAC-IP3R3 interaction in both control and *PRKN*-mutant patient lines (Figures 2A–C; control, *p* < 0.0001; PRKN, *p* < 0.0001), similar to the results of CLEM analysis. Comparing control and *PRKN*-mutant patient lines, PLA in dopaminergic neurons under normal conditions showed that the *PRKN*-mutant patient lines had significantly less VDAC-IP3R3 interaction than the control lines (Figures 2A, C; *p* < 0.0001). Furthermore, we found that the VDAC-IP3R3 interaction was significantly reduced in dopaminergic neurons from *PRKN*-mutant patients compared to control dopaminergic neurons under CCCP treatment (Figures 2B, C; *p* = 0.0029). The difference in the amount of VDAC-IP3R3 interaction between PB2 T1-1 and PB2 T1-4 lines both under normal conditions and CCCP treatment (Figures 2A–C) may possibly be due to the difference in the gene expression specific to each cell lines.

To investigate whether the reduced VDAC-IP3R3 interaction in *PRKN*-mutant patient lines results from a reduction in the amount of mitochondria and/or ER, we stained the *TH*-GFP iPSC-derived neurons with anti-GFP, anti-Tom20, and anti-Calnexin antibodies. Immunofluorescence staining showed that the immunoreactivity for calnexin in *PRKN*-mutant lines was not decreased compared to that in control lines (Supplementary Figures S1A–C). Additionally, the immunoreactivity for Tom20 in *PRKN*-mutant lines was rather increased compared to that in control lines under normal conditions (Supplementary Figures S1A, C; *p* = 0.0029). However, in *PRKN*-mutant patient lines the immunoreactivity for Tom20 under CCCP treatment was decreased compared to that under normal conditions (Supplementary Figures S1A–C; *p* < 0.0001), suggesting that the decrease in VDAC-IP3R3 interaction in *PRKN*-mutant patient lines under CCCP treatment is probably due to the decrease in the amount of mitochondria.

Taken together, these results suggest that *PRKN*-mutant patient dopaminergic neurons derived from *TH*-GFP iPSCs have fewer ER-mitochondria interactions than control dopaminergic neurons under normal conditions, and the reduction of ERMCS in *PRKN*-mutant patient dopaminergic neurons is exacerbated by CCCP treatment.

### PLA for the ERMCS in dopaminergic neurons derived from other iPSC lines

**FIGURE 3:**
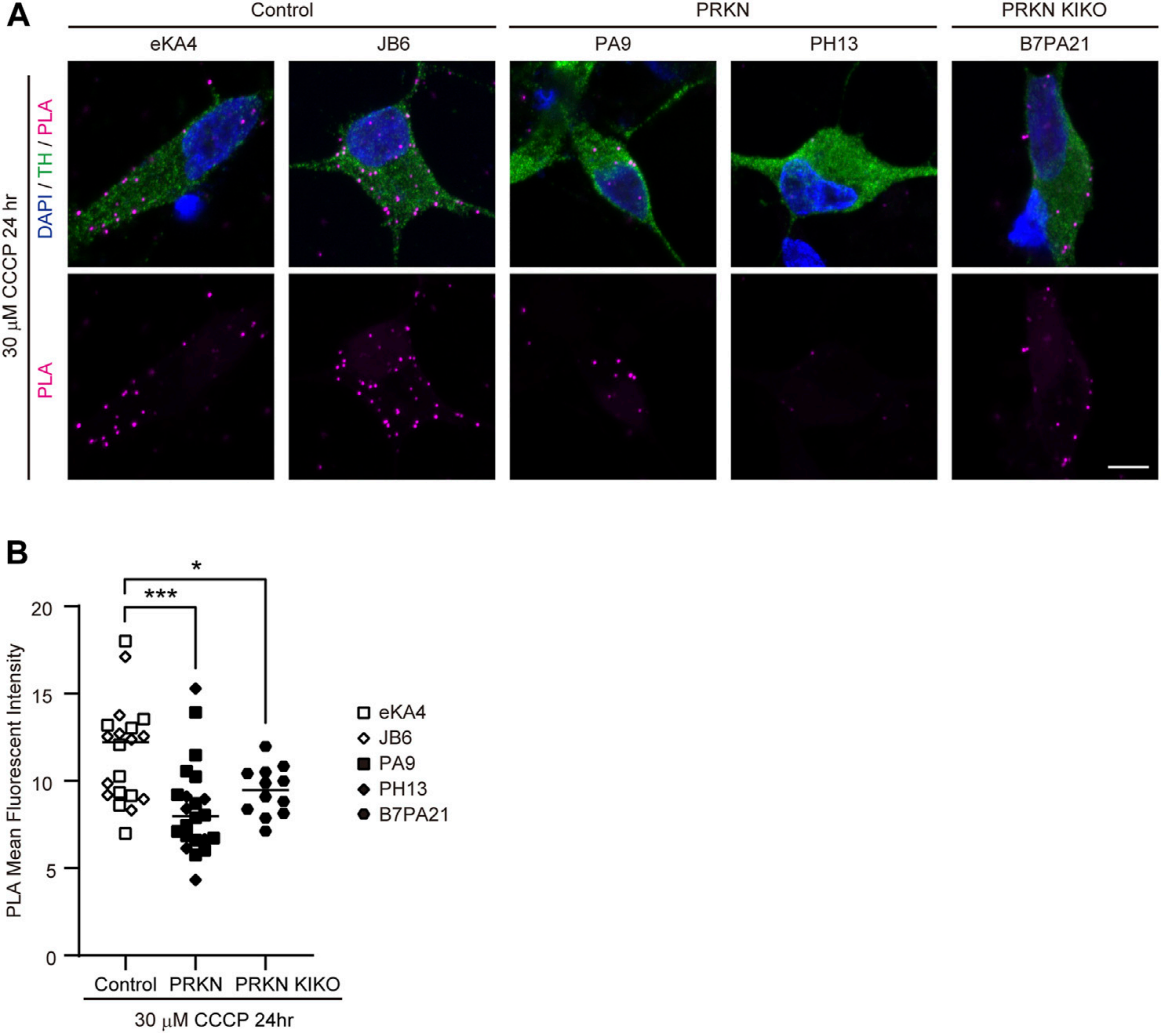
PLA in dopaminergic neurons derived from other iPSCs. **(A)** Representative PLA images of TH-positive dopaminergic neurons under 30 μM CCCP treatment for 24 h derived from other iPSCs. PLA signals represent the interactions between VDAC and IP3R3. “PRKN” and “PRKN-KIKO” represents *PRKN*-mutant patient and *PRKN* knock-in/knock-out, respectively. Scale bar, 5 µm. **(B)** Quantitative analysis of the MFI of PLA signals in TH-positive dopaminergic neurons in PLA images. The MFI was measured by surrounding the cell body region of TH-positive cells (control; n = 20, PRKN; n = 22, PRKN KIKO; n = 12) in ZEN software. “PRKN” and “PRKN-KIKO” represents *PRKN*-mutant patient and *PRKN* knock-in/knock-out, respectively. Horizontal lines indicate median values. Statistical significance was evaluated using the two-way ANOVA with Bonferroni’s multiple comparisons test. *p < 0.05, ***p < 0.001.

To verify whether other patients with *PRKN* mutations or *PRKN*-deficient cells also have a reduced ERMCS, we differentiated into dopaminergic neurons from two control iPSC lines (eKA4 and JB6) and two patient iPSC lines with *PRKN* mutations (PH13 and PA9), and a *PRKN* knock-in/knock-out iPSC line (B7/PA21) (Imaizumi et al., 2012; Matsumoto et al., 2016; Kuzumaki et al., 2019). The PLA in dopaminergic neurons under 30 μM CCCP treatment for 24 h demonstrated that the VDAC-IP3R3 interactions were markedly reduced in the PA9 and PH13 lines compared with the eKA4 and JB6 lines (Figures 3A, B; *p* = 0.0004), indicating that ER-mitochondria interactions were also reduced in dopaminergic neurons derived from other patients with *PRKN* mutations. Similarly, the PLA using the B7/PA21 line showed a reduction of the VDAC-IP3R3 signals in *PRKN* knock-in/knock-out dopaminergic neurons under CCCP treatment (Figures 3A, B; *p* = 0.0467), suggesting that the loss of Parkin resulted in the reduction of ERMCS.

### Mitochondrial Ca^2+^ imaging in GFP-positive dopaminergic neurons derived from *TH*-GFP iPSCs

**FIGURE 4:**
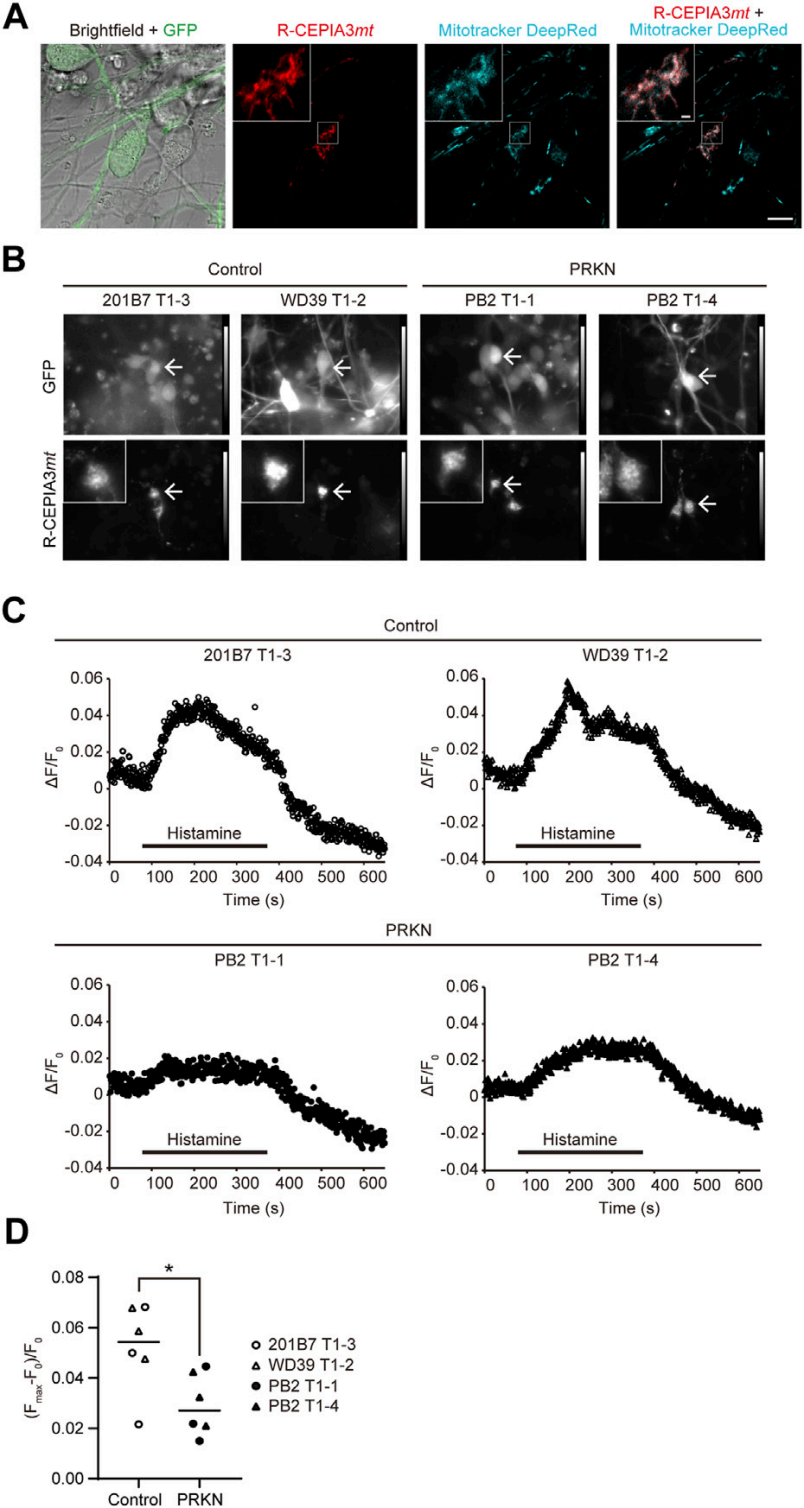
Mitochondrial Ca^2+^ imaging in GFP-positive dopaminergic neurons derived from *TH*-GFP iPSCs. **(A)** Live cell images of R-CEPIA3*mt*-expressing WD39 T1-2 dopaminergic neurons stained with Mitotracker DeepRed. Scale bar, 10 µm. The boxed areas are enlarged to show the cytoplasmic area of GFP-positive R-CEPIA3*mt*-expressing dopaminergic neurons. Scale bar of the enlarged images, 1 µm.**(B)** Representative GFP and R-CEPIA3*mt* images of the differentiated cells derived from control and *PRKN*-mutant patient *TH*-GFP iPSCs before mitochondrial Ca^2+^ measurement. Arrows indicate GFP-positive R-CEPIA3*mt*-expressing dopaminergic neurons. “PRKN” represents *PRKN*-mutant patient. The boxed areas are enlarged to show the cytoplasmic area of GFP-positive R-CEPIA3*mt*-expressing dopaminergic neurons. **(C)** Representative time course of the ratio of the change in R-CEPIA3*mt* fluorescence intensity after histamine treatment in GFP-positive dopaminergic neurons derived from *TH*-GFP iPSCs. ΔF/F_0_ = (fluorescence intensity—minimum fluorescence intensity before stimulation)/minimum fluorescence intensity before stimulation. “PRKN” represents *PRKN*-mutant patient. **(D)** Quantitative analysis of maximum Ca^2+^ flux in control and *PRKN*-mutant dopaminergic neurons derived from *TH*-GFP iPSCs. (F_max_–F_0_)/F_0_ = (maximum fluorescence intensity during stimulation—minimum fluorescence intensity before stimulation)/minimum fluorescence intensity before stimulation. The quantitation was performed for six control dopaminergic neurons and six *PRKN*-mutant dopaminergic neurons, with a ROI surrounding the cytoplasmic area. “PRKN” represents *PRKN*-mutant patient. Statistical significance was evaluated using the unpaired *t*-test. Horizontal lines indicate median values. *p < 0.05.

The ERMCS has been reported to be associated with calcium flux from the ER into the mitochondria (Bravo et al., 2011; Rowland and Voeltz, 2012; Krols et al., 2016; Paillusson et al., 2016; Gómez-Suaga et al., 2018). To investigate whether mitochondrial Ca^2+^ flux is reduced due to decreased ERMCS in dopaminergic neurons from *PRKN*-mutant patients, we used the red fluorescent genetically encoded mitochondrial Ca^2+^ indicator R-CEPIA3*mt* (Kanemal et al., 2020). The CEPIA3*mt* can be used for concentration-dependent intramitochondrial Ca^2+^ imaging (Suzuki et al., 2014; Kanemal et al., 2020). The lentivirus expressing R-CEPIA3*mt* was infected into the cells on day 1 after replating dissociated neurospheres derived from the *TH-*GFP iPSCs. On day 8 after infection, the localization of R-CEPIA3*mt* to mitochondria in GFP-positive dopaminergic neurons was confirmed by staining the differentiated cells with Mitotracker Deep Red (Figure 4A). The signals of R-CEPIA3*mt* fluorescence were detected and the intensity of that was measured in GFP-positive dopaminergic neurons derived from all the *TH*-GFP iPSC lines for mitochondrial Ca^2+^ imaging (Figures 4B, C).

To detect the mitochondrial Ca^2+^ flux, we stimulated cells with histamine, an inositol triphosphate-generating agonist for Ca^2+^ release from the ER to the mitochondria. Histamine stimulation induced an increase in R-CEPIA3*mt* fluorescence intensity in control GFP-positive dopaminergic neurons, whereas the ratio of change in fluorescence intensity in *PRKN*-mutant dopaminergic neurons was small compared to that in control lines. (Figures 4C, D; *p* = 0.0252). To exclude the possibility that the decreased calcium flux to the mitochondria in *PRKN*-mutant patient lines depends on cytosolic Ca^2+^ levels, we stained the *TH*-GFP iPSC-derived neurons with cytosolic Ca^2+^ fluorescent indicator Fura 2-AM and measured the Fura 2-AM fluorescence ratio under histamine stimulation. Cytosolic Ca^2+^ imaging showed no significant difference in cytosolic Ca^2+^ levels between control and *PRKN*-mutant patient lines under histamine stimulation (Supplementary Figures S2A, B; *p* = 0.2037). These findings suggest that the failure of calcium flux from the ER to the mitochondria is not due to low concentrations of cytosolic Ca^2+^ in *PRKN*-mutant dopaminergic neurons. These findings raise the possibility that the impaired ERMCS may be partly responsible for the death of dopaminergic neurons in patients with *PRKN* mutations.

## Discussion

In this study, we showed that CCCP treatment induced a decrease in ERMCS compared to normal conditions in *TH*-GFP iPSC-derived dopaminergic neurons using CLEM and PLA. It is known that CCCP treatment induces Parkin to ubiquitinate Mfn2, a tethering protein at ERMCS. It was reported that Parkin-mediated ubiquitinated Mfn2 was retrotranslocated from the outer mitochondrial membrane, and that ERMCS was decreased during CCCP-induced mitophagy (McLelland et al., 2018). Further studies on non-ubiquitinated Mfn2 in *PRKN*-mutant dopaminergic neurons under CCCP treatment are needed to clarify the effect of Parkin deficiency on ERMCS under pathological conditions.

We also demonstrated reduced ERMCS in *PRKN*-mutant patient dopaminergic neurons derived from *TH-*GFP iPSCs under both normal conditions and CCCP treatment using PLA. In addition, we performed the quantitative analysis of ERMCS in EM images according to the methods used in previous studies (Watanabe et al., 2016; Paillusson et al., 2017; Parrado-Fernández et al., 2018; Liu et al., 2019). Our CLEM analysis did not show significant difference in ERMCS between control and *PRKN*-mutant patient dopaminergic neurons. This discrepancy between CLEM and PLA might be due to the difference between the two-dimensional analysis using ultrathin sections and the three-dimensional analysis using whole cells. Consistent with our results in PLA, a previous study has reported the reduction of ERMCS in Parkin downregulating mouse embryonic fibroblasts and *PRKN*-mutant patient fibroblasts (Basso et al., 2018). Furthermore, several studies on other PD-causing proteins have reported reduced ERMCS in iPSC-derived dopaminergic neurons with triplication of the α-synuclein gene (Paillusson et al., 2017), PINK1 or DJ-1 knockdown neuroblastoma cells (Parrado-Fernández et al., 2018), and DJ-1 knockout neuroblastoma cells (Liu et al., 2019). However, some studies have reported increased ERMCS in *PRKN*-mutant patient fibroblasts (Gautier et al., 2016) and *PRKN*-mutant dopaminergic neurons (McLelland et al., 2018). Whether ERMCS are increased or decreased in PD patients is still controversial.

PLA in *PRKN* knock-in/knock-out dopaminergic neurons indicated that the loss of Parkin resulted in the reduction of ERMCS. Our results are consistent with those of a previous study, which reported that silencing of Parkin in SH-SY5Y cells impairs ERMCS and Ca^2+^ transients (Calì et al., 2013). It has been suggested that Parkin is present at ERMCS and interacts with other ERMCS-associated proteins (Gelmetti et al., 2017). Thus, Parkin deficiency may disrupt ERMCS in dopaminergic neurons.

PLA is a highly sensitive method, but it relies on the specificity of individual antibodies against endogenous proteins. Non-specific binding of the antibodies causes a high false positive rate (Wilson and Metzakopian, 2021). The use of other methods such as split fluorescence reporters of ERMCS in future studies would be valuable for the more precise validation of ERMCS.

Mitochondrial Ca^2+^ imaging showed that the mitochondrial Ca^2+^ flux was reduced in *PRKN*-mutant dopaminergic neurons after histamine-induced Ca^2+^ release from the ER to mitochondria, suggesting impaired ERMCS-mediated Ca^2+^ flux from the ER to mitochondria. Our results are consistent with those of several previous studies that reported decreased mitochondrial Ca^2+^ uptake in *PRKN*-mutant patient fibroblasts (Basso et al., 2018) and DJ-1 knockout cells (Liu et al., 2019). The previous study also demonstrated decreased ATP production in DJ-1 knockout cells, suggesting mitochondrial dysfunction (Liu et al., 2019). In addition, it has been reported that dopaminergic neurons from *PRKN*-mutant patients show a failure of mitochondrial degradation (Imaizumi et al., 2012; Suzuki et al., 2017; Yamaguchi et al., 2020). The defects and mitochondrial Ca^2+^ flux in dopaminergic neurons from *PRKN*-mutant patients in our study may lead to the accumulation of damaged mitochondria and subsequent cell death of *PRKN*-mutant dopaminergic neurons. Expression of a linker or treatment with an agonist for the ERMCS could clarify whether the reduced ERMCS affects the cell death of *PRKN*-mutant dopaminergic neurons under CCCP treatment.

On the other hand, cytosolic Ca^2+^ levels were not increased after histamine stimulation despite decreased mitochondrial Ca^2+^ flux in *PRKN*-mutant dopaminergic neurons. A possible cause for this is the decrease in Ca^2+^ release from the ER in *PRKN*-mutant patient dopaminergic neurons. The Ca^2+^ release from the ER could not be measured in this study, but the measurement of that in *PRKN*-mutant patient dopaminergic neurons is necessary to be addressed in further studies.

In the present study, we first visualized the mitochondrial Ca^2+^ flux specific to dopaminergic neurons using *PRKN*-mutant patient *TH*-GFP iPSC lines and R-CEPIA3*mt*. Our strategy of using *TH*-GFP iPSCs overcame the heterogeneity of iPSC-derived differentiated cells composed of dopaminergic and non-dopaminergic neurons in mitochondrial Ca^2+^ imaging. However, the limitation of our study is the small number of *TH*-GFP iPSC lines used for CLEM and mitochondrial Ca^2+^ imaging. Future studies with other *TH-*GFP iPSC lines are needed to clarify the general differences between the control and *PRKN*-mutant patients.

In conclusion, PLA using *TH-*GFP iPSC lines revealed reduced ERMCS in *PRKN*-mutant dopaminergic neurons under both normal conditions and CCCP treatment. Furthermore, mitochondrial Ca^2+^ imaging demonstrated a decreased mitochondrial Ca^2+^ flux in *PRKN*-mutant dopaminergic neurons, suggesting impaired Ca^2+^ flux from the ER to mitochondria. Our findings would provide insights into the pathogenesis of cell death in dopaminergic neurons in *PRKN*-mutant patients.

## Data Availability

The raw data supporting the conclusion of this article will be made available by the authors, without undue reservation.

## Supplementary material

SUPPLEMENTARY FIGURE S1 Immunofluorescence staining with anti-Tom20 and anti-Calnexin antibodies in dopaminergic neurons derived from TH-GFPiPSCs. **(A)** Representative fluorescence images of dopaminergic neurons under normal conditions derived from control and PRKN-mutant patient TH-GFP iPSCs. “PRKN” represents PRKN-mutant patient. Scale bar, 5 µm. **(B)** Representative fluorescence images of dopaminergic neurons under 30 μM CCCP treatment for 24 hours derived from control and PRKN-mutant patient TH-GFP iPSCs. “PRKN” represents PRKN-mutant patient. Scale bar, 5 µm. **(C)** Quantitative analysis of the percentage of Tom20 (left) and Calnexin (right) staining positive area in dopaminergic neurons from fluorescence images. The staining positive areas were measured using the ImageJ threshold tool in manually surrounding the area of GFP-positive cells (DMSO control; n = 10, DMSO PRKN; n = 10, CCCP control; n = 6, CCCP PRKN; n = 7). “PRKN” represents PRKN-mutant patient. Horizontal lines indicate median values. Statistical significance was evaluated using the two-way ANOVA with Bonferroni’s multiple comparisons test. **P < 0.01, ****P < 0.0001. There was no significant difference in staining area between DMSO and CCCP in control(Tom20; P = 0.2584, Calnexin; P = 0.0777) and PRKN-mutant (Calnexin; P = 0.0969) dopaminergic neurons. There was no significant difference in staining area between control and PRKN-mutant patient lines under normal conditions (Calnexin; P = 0.1995) and CCCP treatment (Tom20; P = 0.8349, Calnexin; P = 0.4927).

SUPPLEMENTARY FIGURE S2 Cytosolic Ca2+ imaging in GFP-positive dopaminergic neurons derived from TH-GFP iPSCs. **(A)** Representative time course of the ratio of the change in the Fura 2-AM fluorescence ratio after histamine treatment in GFP-positive dopaminergic neurons derived from TH-GFP iPSCs. ΔR/R0 = (fluorescence ratio – minimum fluorescence ratio before stimulation) / minimum fluorescence ratio before stimulation. “PRKN” represents PRKN-mutant patient. **(B)** Quantitative analysis of maximum Ca^2+^ flux in control and PRKN-mutant dopaminergic neurons derived from TH-GFP iPSCs. (Rmax–R0)/R0 = (maximum fluorescence ratio during stimulation – minimum fluorescence ratio before stimulation) / minimum fluorescence ratio before stimulation. The quantitation was performed for eight control dopaminergic neurons and eight PRKN-mutant dopaminergic neurons, with a ROI surrounding the cytoplasmic area. “PRKN” represents PRKN-mutant patient. Statistical significance was evaluated using the unpaired t-test. Horizontal lines indicate median values. There was no significant difference in (Rmax–R0)/R0 between control and PRKN-mutant patient dopaminergic neurons (P = 0.2037).

## References

[B1] Abou-Sleiman P. M. Muqit M. M. Wood N. W. (2006). Expanding insights of mitochondrial dysfunction in Parkinson’s disease. Nat. Rev. Neurosci. 7, 207–219. 10.1038/nrn1868 16495942

[B2] Basso V. Marchesan E. Peggion C. Chakraborty J. Von Stockum S. Giacomello M. (2018). Regulation of ER-mitochondria contacts by parkin via Mfn2. Pharmacol. Res. 138, 43–56. 10.1016/j.phrs.2018.09.006 30219582

[B3] Bravo R. Vicencio J. M. Parra V. Troncoso R. Munoz J. P. Bui M. (2011). Increased ER-mitochondrial coupling promotes mitochondrial respiration and bioenergetics during early phases of ER stress. J. Cell Sci. 124, 2143–2152. 10.1242/jcs.080762 21628424 PMC3113668

[B4] Calì T. Ottolini D. Negro A. Brini M. (2013). Enhanced parkin levels favor ER-mitochondria crosstalk and guarantee Ca^2+^ transfer to sustain cell bioenergetics. Biochim. Biophys. Acta 1832, 495–508. 10.1016/j.bbadis.2013.01.004 23313576

[B5] Csordás G. Weaver D. Hajnóczky G. (2018). Endoplasmic reticulum-mitochondrial contactology: structure and signaling functions. Trends Cell Biol. 28, 523–540. 10.1016/j.tcb.2018.02.009 29588129 PMC6005738

[B6] Fujimori K. Matsumoto T. Kisa F. Hattori N. Okano H. Akamatsu W. (2017). Escape from pluripotency via inhibition of TGF-β/BMP and activation of Wnt signaling accelerates differentiation and aging in hPSC progeny cells. Stem Cell Rep. 9, 1675–1691. 10.1016/j.stemcr.2017.09.024 PMC5831048 29107593

[B7] Gautier C. A. Erpapazoglou Z. Mouton-Liger F. Muriel M. P. Cormier F. Bigou S. (2016). The endoplasmic reticulum-mitochondria interface is perturbed in PARK2 knockout mice and patients with PARK2 mutations. Hum. Mol. Genet. 25, 2972–2984. 10.1093/hmg/ddw148 27206984

[B8] Gelmetti V. De Rosa P. Torosantucci L. Marini E. S. Romagnoli A. Di Rienzo M. (2017). PINK1 and BECN1 relocalize at mitochondria-associated membranes during mitophagy and promote ER-mitochondria tethering and autophagosome formation. Autophagy 13, 654–669. 10.1080/15548627.2016.1277309 28368777 PMC5388214

[B9] Gómez-Suaga P. Bravo-San Pedro J. M. González-Polo R. A. Fuentes J. M. Niso-Santano M. (2018). ER-mitochondria signaling in Parkinson’s disease. Cell Death Dis. 9, 337. 10.1038/s41419-017-0079-3 29497039 PMC5832754

[B10] Guardia-Laguarta C. Area-Gomez E. Rüb C. Liu Y. Magrané J. Becker D. (2014). α-Synuclein is localized to mitochondria-associated ER membranes. J. Neurosci. 34, 249–259. 10.1523/JNEUROSCI.2507-13.2014 24381286 PMC3866487

[B11] Hedskog L. Pinho C. M. Filadi R. Rönnbäck A. Hertwig L. Wiehager B. (2013). Modulation of the endoplasmic reticulum-mitochondria interface in Alzheimer’s disease and related models. Proc. Natl. Acad. Sci. U. S. A. 110, 7916–7921. 10.1073/pnas.1300677110 23620518 PMC3651455

[B12] Imaizumi K. Sone T. Ibata K. Fujimori K. Yuzaki M. Akamatsu W. (2015). Controlling the regional identity of hPSC-derived neurons to uncover neuronal subtype specificity of neurological disease phenotypes. Stem Cell Rep. 5, 1010–1022. 10.1016/j.stemcr.2015.10.005 PMC4682123 26549851

[B13] Imaizumi Y. Okada Y. Akamatsu W. Koike M. Kuzumaki N. Hayakawa H. (2012). Mitochondrial dysfunction associated with increased oxidative stress and alpha-synuclein accumulation in PARK2 iPSC-derived neurons and postmortem brain tissue. Mol. Brain. 5, 35. 10.1186/1756-6606-5-35 23039195 PMC3546866

[B14] Ishikawa K. I. Ishiguro M. Li Y. Nishioka K. Hattori N. Akamatsu W. (2022). Generation of three hiPSC clones from a Parkinson's disease patient with a heterozygous variant of VPS35 p.D620N. Stem. Cell. Res. 60, 102739. 10.1016/j.scr.2022.102739 35247840

[B15] Kanemal K. Suzuki J. Taiko I. Iino M. (2020). Red fluorescent CEPIA indicators for visualization of Ca^2+^ dynamics in mitochondria. Sci. Rep. 10, 2835. 10.1038/s41598-020-59707-8 32071363 PMC7029041

[B16] Krols M. Van Isterdael G. Asselbergh B. Kremer A. Lippens S. Timmerman V. (2016). Mitochondria-associated membranes as hubs for neurodegeneration. Acta Neuropathol. 131, 505–523. 10.1007/s00401-015-1528-7 26744348 PMC4789254

[B17] Kuzumaki N. Suda Y. Iwasawa C. Narita M. Sone T. Watanabe M. (2019). Cell-specific overexpression of COMT in dopaminergic neurons of Parkinson’s disease. Brain 142, 1675–1689. 10.1093/brain/awz084 31135049

[B18] Liu Y. Ma X. Fujioka H. Liu J. Chen S. Zhu X. (2019). DJ-1 regulates the integrity and function of ER-mitochondria association through interaction with IP3R3-Grp75-VDAC1. Proc. Natl. Acad. Sci. U. S. A. 116, 25322–25328. 10.1073/pnas.1906565116 31767755 PMC6911199

[B19] Matsumoto T. Fujimori K. Andoh-Noda T. Ando T. Kuzumaki N. Toyoshima M. (2016). Functional neurons generated from T cell-derived induced pluripotent stem cells for neurological disease modeling. Stem Cell Rep. 6, 422–435. 10.1016/j.stemcr.2016.01.010 PMC4788773 26905201

[B20] Mclelland G. L. Goiran T. Yi W. Dorval G. Chen C. X. Lauinger N. D. (2018). Mfn2 ubiquitination by PINK1/parkin gates the p97-dependent release of ER from mitochondria to drive mitophagy. eLife 7, e32866. 10.7554/eLife.32866 29676259 PMC5927771

[B21] Narendra D. Tanaka A. Suen D. F. Youle R. J. (2008). Parkin is recruited selectively to impaired mitochondria and promotes their autophagy. J. Cell Biol. 183, 795–803. 10.1083/jcb.200809125 19029340 PMC2592826

[B22] Okano H. Morimoto S. (2022). iPSC-based disease modeling and drug discovery in cardinal neurodegenerative disorders. Cell Stem Cell 29, 189–208. 10.1016/j.stem.2022.01.007 35120619

[B23] Ottolini D. Calì T. Negro A. Brini M. (2013). The Parkinson disease-related protein DJ-1 counteracts mitochondrial impairment induced by the tumour suppressor protein p53 by enhancing endoplasmic reticulum-mitochondria tethering. Hum. Mol. Genet. 22, 2152–2168. 10.1093/hmg/ddt068 23418303

[B24] Paillusson S. Gomez-Suaga P. Stoica R. Little D. Gissen P. Devine M. J. (2017). α-synuclein binds to the ER-mitochondria tethering protein VAPB to disrupt Ca^2+^ homeostasis and mitochondrial ATP production. Acta Neuropathol. 134, 129–149. 10.1007/s00401-017-1704-z 28337542 PMC5486644

[B25] Paillusson S. Stoica R. Gomez-Suaga P. Lau D. H. W. Mueller S. Miller T. (2016). There’s something wrong with my MAM; the ER-mitochondria axis and neurodegenerative diseases. Trends Neurosci. 39, 146–157. 10.1016/j.tins.2016.01.008 26899735 PMC4780428

[B26] Parrado-Fernández C. Schneider B. Ankarcrona M. Conti M. M. Cookson M. R. Kivipelto M. (2018). Reduction of PINK1 or DJ-1 impair mitochondrial motility in neurites and alter ER-mitochondria contacts. J. Cell. Mol. Med. 22, 5439–5449. 10.1111/jcmm.13815 30133157 PMC6201361

[B27] Rowland A. A. Voeltz G. K. (2012). Endoplasmic reticulum-mitochondria contacts: function of the junction. Nat. Rev. Mol. Cell Biol. 13, 607–625. 10.1038/nrm3440 22992592 PMC5111635

[B28] Scarffe L. A. Stevens D. A. Dawson V. L. Dawson T. M. (2014). Parkin and PINK1: much more than mitophagy. Trends Neurosci. 37, 315–324. 10.1016/j.tins.2014.03.004 24735649 PMC4075431

[B29] Söderberg O. Gullberg M. Jarvius M. Ridderstråle K. Leuchowius K. J. Jarvius J. (2006). Direct observation of individual endogenous protein complexes *in situ* by proximity ligation. Nat. Methods. 3, 995–1000. 10.1038/nmeth947 17072308

[B30] Suzuki J. Kanemaru K. Ishii K. Ohkura M. Okubo Y. Iino M. (2014). Imaging intraorganellar Ca2+ at subcellular resolution using CEPIA. Nat. Commun. 5, 4153. 10.1038/ncomms5153 24923787 PMC4082642

[B31] Suzuki S. Akamatsu W. Kisa F. Sone T. Ishikawa K. I. Kuzumaki N. (2017). Efficient induction of dopaminergic neuron differentiation from induced pluripotent stem cells reveals impaired mitophagy in PARK2 neurons. Biochem. Biophys. Res. Commun. 483, 88–93. 10.1016/j.bbrc.2016.12.188 28057485

[B32] Takahashi K. Tanabe K. Ohnuki M. Narita M. Ichisaka T. Tomoda K. (2007). Induction of pluripotent stem cells from adult human fibroblasts by defined factors. Cell 131, 861–872. 10.1016/j.cell.2007.11.019 18035408

[B33] Watanabe S. Ilieva H. Tamada H. Nomura H. Komine O. Endo F. (2016). Mitochondria-associated membrane collapse is a common pathomechanism in SIGMAR1-and SOD1-linked ALS. EMBO Mol. Med. 8, 1421–1437. 10.15252/emmm.201606403 27821430 PMC5167132

[B34] Wilson E. L. Metzakopian E. (2021). Correction: ER-mitochondria contact sites in neurodegeneration: genetic screening approaches to investigate novel disease mechanisms. Cell Death Differ. 10, 2990. 10.1038/s41418-020-00723-6 PMC8481315 33437039

[B35] Yamaguchi A. Ishikawa K. I. Inoshita T. Shiba-Fukushima K. Saiki S. Hatano T. (2020). Identifying therapeutic agents for amelioration of mitochondrial clearance disorder in neurons of familial Parkinson disease. Stem Cell Rep. 14, 1060–1075. 10.1016/j.stemcr.2020.04.011 PMC7355139 32470327

[B36] Yokota M. Kakuta S. Shiga T. Ishikawa K. I. Okano H. Hattori N. (2021). Establishment of an *in vitro* model for analyzing mitochondrial ultrastructure in PRKN-mutated patient iPSC-derived dopaminergic neurons. Mol. Brain. 14, 58. 10.1186/s13041-021-00771-0 33757554 PMC7986497

