# Supplementary figures and images for: Reduced ER-mitochondrial contact sites and mitochondrial Ca2+ flux in PRKN-mutant patient tyrosine hydroxylase reporter iPSC lines
Source: Front Cell Dev Biol. 2023 Sep 8;11:1171440. doi: 10.3389/fcell.2023.1171440 (PMC10514478; doi:10.3389/fcell.2023.1171440)

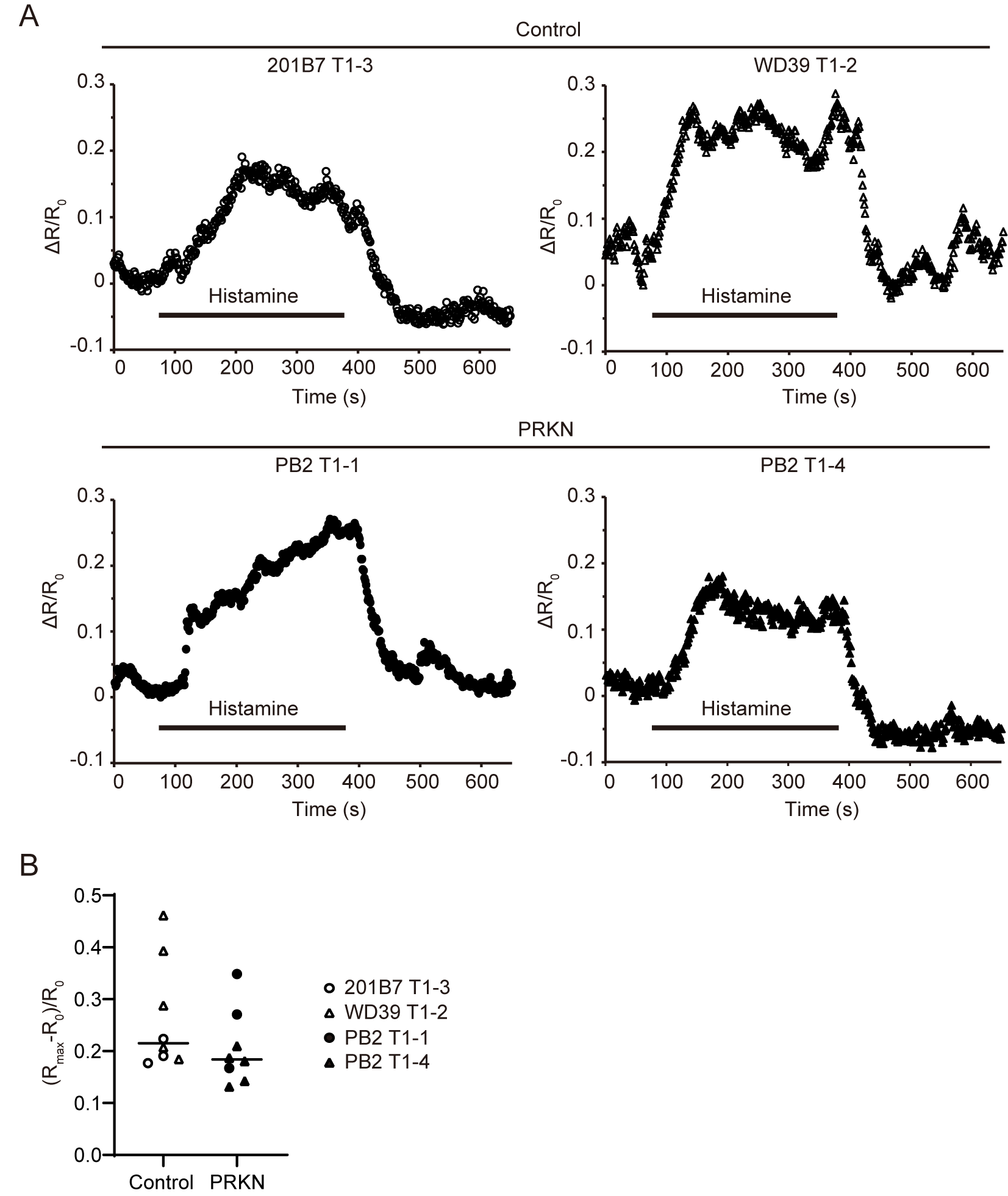

Supplement: Supplementary file 1 [file Image2.TIF]

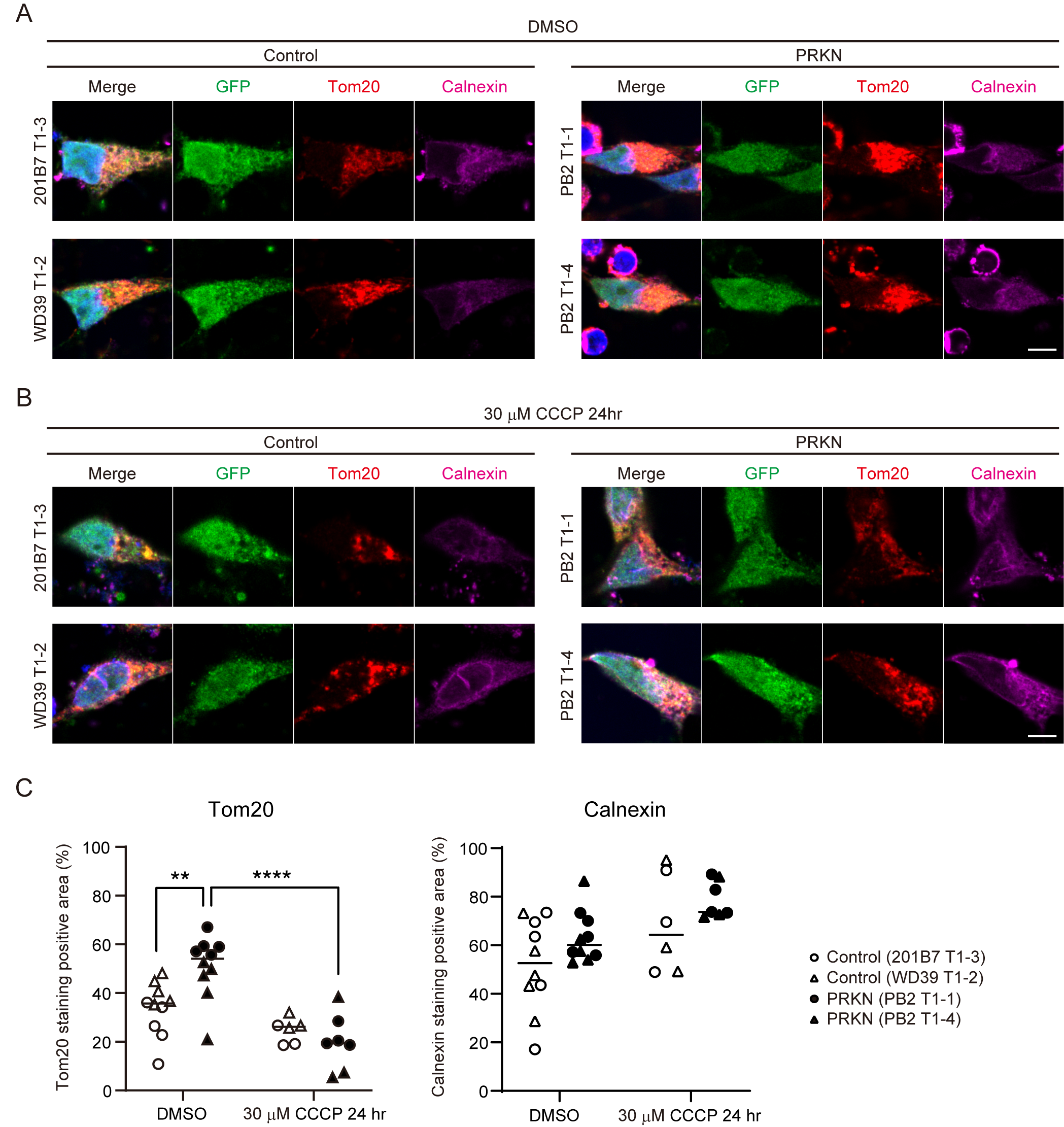

Supplement: Supplementary file 2 [file Image1.TIF]
